# Lumbar Magnetic Resonance Imaging Profiles and Their Association with Low Back Pain in Dentists: A Cluster Analysis Study

**DOI:** 10.3390/diagnostics16152421

**Published:** 2026-07-31

**Authors:** Ana Lopez-Morales, Germán Cánovas-Ambit, Aitor Baño-Alcaraz, Manuel López-Nicolás, Francesc Medina-Mirapeix, J. A. García-Vidal

**Affiliations:** 1 Department of Dermatology, Stomatology, Radiology and Physical Medicine, University of Murcia, 30008 Murcia, Spain; lopezmoralesana0@gmail.com (A.L.-M.); malopez@um.es (M.L.-N.); 2 Department of Physiotherapy, Faculty of Medicine, University of Murcia, Campus de Espinardo, 30100 Murcia, Spain; aitor.bano@um.es (A.B.-A.); mirapeix@um.es (F.M.-M.); garciavidal@um.es (J.A.G.-V.); 3 Biomedical Research Institute of Murcia IMIB-Pascual Parrilla, Campus de Espinardo, 30100 Murcia, Spain

**Keywords:** low back pain, magnetic resonance imaging, cluster analysis, dentists, intervertebral disc degeneration

## Abstract

**Background/Objectives:** Low back pain (LBP) is highly prevalent among dentists, although the relationship between lumbar magnetic resonance imaging (MRI) findings and symptoms remains unclear. This study aimed to identify lumbar MRI-derived structural profiles in actively practicing dentists and to examine their relationship with LBP, pain-related disability, demographic characteristics, occupational factors, and individual MRI findings. **Methods:** A cross-sectional observational study was conducted in 57 actively practicing dentists. Demographic, occupational, and clinical data were collected, and lumbar MRI examinations were performed using standardized protocols. MRI findings included disc bulging, disc herniation, annular fissure, Modic changes, osteophytes, facet joint degeneration, and spinal canal stenosis. Cluster analysis was used to identify MRI-derived structural profiles. Associations between profiles, LBP, disability, demographic characteristics, occupational factors, and individual MRI findings were assessed using chi-square tests and correspondence analysis. **Results:** Two distinct MRI-derived structural profiles were identified: a disc–facet degenerative profile (*n* = 38) and a non-osseous degeneration profile (*n* = 19). Disc bulging (100% vs. 52.6%, *p* < 0.001), facet joint degeneration (94.7% vs. 10.5%, *p* < 0.001), and osteophytes (23.7% vs. 0%, *p* = 0.022) significantly contributed to profile differentiation, whereas disc herniation, Modic changes, and annular fissure did not. Neither MRI-derived profile was associated with the presence of LBP or pain-related disability (*p* > 0.05). Younger dentists were more frequently classified within the disc–facet degenerative profile with LBP, whereas older dentists with similar structural findings were more commonly classified within the same profile without LBP (*p* = 0.007). No significant associations were observed between MRI-derived profiles and occupational characteristics. **Conclusions:** Two distinct lumbar MRI-derived structural profiles were identified in dentists, but neither was associated with LBP or pain-related disability. Age appeared to influence the clinical expression of the disc–facet degenerative profile, suggesting that lumbar MRI findings should be interpreted within a broader, age-sensitive clinical context rather than as isolated determinants of symptoms.

## 1. Introduction

Low back pain (LBP) is a major public health problem and remains one of the most common causes of disability worldwide. It is estimated that up to 80% of individuals will experience at least one episode of LBP during their lifetime [1,2]. Beyond its high prevalence, LBP imposes a substantial socioeconomic burden due to healthcare costs, reduced work productivity, absenteeism, and diminished quality of life. It represents one of the leading causes of years lived with disability and has become an important occupational health concern across a wide range of professions [1,2]. Healthcare professionals are particularly susceptible to this condition because their daily activities often involve prolonged static postures, repetitive movements, sustained physical demands, and inadequate ergonomic conditions [3]. Among these professionals, dentists consistently report one of the highest prevalences of LBP, with published estimates ranging between 62% and 93% [4,5,6]. The nature of dental practice frequently requires prolonged forward trunk flexion, neck inclination, repetitive fine motor tasks, and maintenance of constrained working postures for extended periods. Over time, these occupational exposures may contribute to cumulative mechanical stress on the lumbar spine and increase the risk of developing musculoskeletal disorders. In addition to their clinical consequences, musculoskeletal disorders may negatively affect dentists’ professional performance, reduce productivity, and contribute to work absenteeism or even early retirement. Consequently, identifying factors associated with lumbar degeneration and LBP in this occupational group remains an important priority for occupational health research. Previous research has identified several factors contributing to this high burden, including age, occupational and ergonomic exposures, as well as degenerative alterations of the lumbar spine [7,8,9,10,11,12].

Magnetic resonance imaging (MRI) is currently considered the reference imaging technique for evaluating degenerative disorders of the lumbar spine because of its ability to accurately detect structural abnormalities [13,14,15]. MRI provides excellent visualization of intervertebral discs, vertebral endplates, facet joints, ligaments, and neural structures without exposing patients to ionizing radiation, making it particularly valuable for the assessment of lumbar degenerative changes. Numerous investigations have assessed the relationship between MRI findings and LBP in both the general population and occupational groups exposed to considerable postural stress [16,17,18]. Nevertheless, the available evidence remains inconclusive [19,20,21]. Although some symptomatic individuals present marked degenerative abnormalities, such as disc herniation or disc bulging, similar imaging findings are also frequently observed in people who do not report low back pain. This lack of a consistent relationship between structural abnormalities and symptoms has generated increasing interest in identifying imaging patterns that may better reflect the complexity of lumbar degeneration than isolated MRI findings.

This apparent discrepancy indicates that isolated MRI abnormalities may have limited value for explaining the clinical presentation of LBP. Because several degenerative changes commonly coexist within the same individual, evaluating the overall pattern of structural abnormalities may provide more clinically relevant information than assessing each imaging finding separately. In this context, multivariate approaches such as cluster analysis have gained increasing attention as useful tools for identifying subgroups of individuals sharing similar structural characteristics [22,23]. By integrating multiple variables simultaneously, this methodology facilitates the identification of clinically meaningful profiles that may reflect distinct pathophysiological processes and improve disease characterization and risk stratification [23]. Furthermore, identifying structural profiles rather than isolated abnormalities may contribute to a more comprehensive understanding of lumbar degeneration and help generate hypotheses regarding its clinical relevance in specific occupational populations.

To the best of our knowledge, lumbar MRI-derived structural profiles have not previously been investigated in dentists using a cluster analysis approach. Moreover, it remains unclear whether combinations of coexisting degenerative abnormalities show a stronger relationship with LBP than isolated MRI findings in this occupational population. Therefore, the objective of the present study was to identify lumbar MRI-derived structural profiles among practicing dentists and to investigate their associations with low back pain, pain-related disability, demographic and occupational characteristics, and individual MRI abnormalities.

## 2. Materials and Methods

### 2.1. Study Design and Participants

This cross-sectional observational study included actively practicing dentists affiliated with the Professional College of Dentists and Stomatologists of Murcia. Participant recruitment was conducted between January and June 2025 through the College’s internal communication system, which distributed information about the study and invited voluntary participation. Participation was entirely voluntary, and no financial or professional incentives were offered. All eligible participants underwent the same standardized assessment protocol, including clinical evaluation and lumbar MRI examination, during a single study visit.

Eligible participants were dentists who had been engaged in clinical practice for at least one year. Individuals were excluded if they had undergone previous lumbar spine surgery, presented neurological or systemic disorders, or had any contraindication to magnetic resonance imaging (MRI). These eligibility criteria were established to minimize potential confounding factors that could influence lumbar MRI findings or the clinical presentation of low back pain. A history of low back pain prior to professional practice was not considered an exclusion criterion. Written informed consent was obtained from all participants before study enrollment. The study was approved by the Ethics Committee of the University of Murcia (CEI-4737/2023) and was conducted in accordance with the ethical principles of the Declaration of Helsinki.

### 2.2. Data Collection

All study procedures were completed during a single assessment session at an authorized radiology center. During this visit, demographic information, occupational characteristics, and pain-related variables were recorded before lumbar MRI examinations were performed.

### 2.3. Demographic and Occupational Variables

Demographic and occupational information potentially associated with LBP was obtained using a structured questionnaire. The variables collected included sex (male/female), age (initially recorded as a continuous variable and subsequently categorized into <40, 40–49, and ≥50 years), years of professional practice, weekly workload (<36 or ≥36 h), predominant working posture (sitting, standing, or alternating between both), and the type of chair most commonly used during clinical practice (without armrests, with one armrest, or with two armrests).

### 2.4. Pain Assessment and MRI Evaluation

The presence of low back pain (LBP) and pain-related disability was evaluated in all participants. LBP was determined by asking whether lumbar pain had been experienced during or after professional practice (yes/no). Disability associated with LBP was assessed using the Oswestry Disability Index (ODI), considering scores greater than 20 as indicative of clinically relevant disability [24].

Lumbar MRI examinations were performed using a 1.5-T General Electric Signa HDxt scanner (GE Healthcare, Milwaukee, WI, USA). Standard lumbar spine imaging protocols were applied, including sagittal and axial T1-weighted and T2-weighted sequences. The imaging assessment comprised the presence of disc bulging, disc herniation, annular fissure, Modic changes, osteophytes, facet joint degeneration, and spinal canal stenosis. All MRI examinations were independently reviewed by an experienced musculoskeletal radiologist with more than five years of clinical experience. The radiologist was blinded to all demographic, occupational, and clinical information. Structural abnormalities were documented according to routine radiological assessment criteria used in daily clinical practice.

### 2.5. Statistical Analysis

Descriptive statistics were used to summarize participant characteristics. Continuous variables are presented as mean ± standard deviation (SD), whereas categorical variables are reported as frequencies and percentages. Normality of continuous variables was assessed prior to analysis. Comparisons between participants with and without low back pain (LBP) were performed using Student’s t-test for continuous variables and chi-square tests for categorical variables.

Cluster analysis was selected because lumbar degenerative abnormalities frequently coexist within the same individual, making it difficult to interpret the clinical relevance of isolated MRI findings. This unsupervised multivariate technique allows participants to be grouped according to the overall pattern of structural abnormalities rather than individual imaging variables, thereby facilitating the identification of clinically meaningful MRI-derived profiles.

Cluster analysis was conducted to identify groups of participants with similar lumbar MRI patterns. Hierarchical clustering was initially applied to determine the optimal number of clusters using squared Euclidean distance, Ward’s method, and Complete Linkage. The final cluster solution was selected according to the dendrogram and agglomeration coefficients, after which participants were automatically grouped according to the similarity of their lumbar MRI findings using a non-hierarchical clustering procedure. Only MRI variables were included in the clustering process, whereas clinical, demographic, and occupational variables were not used to generate the clusters and were analysed only after cluster allocation. The resulting cluster solution was subsequently used as the main categorical variable in the comparative analyses to investigate its relationship with clinical, demographic, and occupational characteristics. Associations between MRI-derived structural profiles, LBP status, and participant subgroups were evaluated using chi-square tests. Correspondence analysis was performed to examine the relationships between age categories and the MRI-derived profile/pain subgroups. Statistical significance was set at *p* < 0.05. All analyses were performed using IBM SPSS Statistics for Windows, version 28.0 (IBM Corp., Armonk, NY, USA).

## 3. Results

### 3.1. Participants

A total of 57 dentists were included in the study. All participants completed the full assessment, with no missing data. Table 1 summarizes the demographic, professional, pain, and MRI characteristics of the sample. The mean (SD) professional experience was 18.1 (11.9) years, and 54.4% of the participants were women. Among the MRI abnormalities identified, the most prevalent alterations were disc bulging (84.2%) and facet joint degeneration (66.7%). Overall, 38 participants (66.7%) reported low back pain during or after work activities.

As shown in Table 1, the age distribution differed significantly between participants with and without LBP (*p* = 0.030). Participants with LBP also showed a higher frequency of facet joint degeneration (76.3% vs. 47.4%, *p* = 0.029). No significant differences were observed between groups regarding sex, years of professional experience, weekly working hours, working position, chair support, or the remaining MRI findings.

### 3.2. MRI-Based Clusters and Pain

As shown in Table 2, the distribution of MRI findings allowed the identification of two distinct structural profiles. Cluster 1 included 38 participants, whereas Cluster 2 comprised 19 participants.

**Figure 1:**
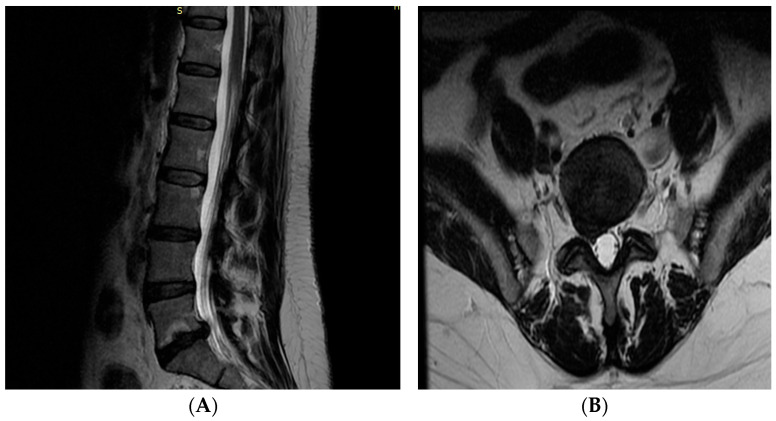
Representative lumbar magnetic resonance imaging (MRI) findings from a participant included in the disc–facet degenerative profile. (**A**) Sagittal T2-weighted image demonstrating multilevel degenerative disc disease with loss of disc signal intensity and posterior disc bulging predominantly involving the lower lumbar spine. (**B**) Corresponding axial T2-weighted image illustrating posterior disc bulging associated with facet joint degeneration. These representative images illustrate the combination of structural abnormalities that characterized the disc–facet degenerative profile identified in the present study.

Almost all participants in Cluster 1 presented facet joint degeneration (94.7%) and disc bulging (100%), defining a disc–facet degenerative profile. Representative MRI findings corresponding to the disc–facet degenerative profile are shown in Figure 1. The images illustrate the coexistence of degenerative disc changes and posterior element degeneration, providing a visual example of the structural abnormalities most frequently identified in participants assigned to this MRI-derived profile.

In contrast, participants in Cluster 2 exhibited a non-osseous degeneration profile, characterized by a moderate frequency of disc bulging (52.6%), a low prevalence of facet joint degeneration (10.5%) and annular fissure (5.3%), and the absence of osteophytes. Disc bulging, facet joint degeneration, and osteophytes differed significantly between clusters (*p* < 0.05), whereas annular fissure did not.

Conversely, disc herniation and Modic changes did not differ significantly between clusters (*p* > 0.05). These findings were similarly distributed across both profiles, suggesting that they did not contribute to cluster differentiation.

Despite these structural differences, no significant differences were observed between clusters regarding the prevalence of low back pain (73.7% vs. 52.6%; *p* = 0.112) or clinically relevant disability (Oswestry Disability Index ≥ 20: 7.9% vs. 15.8%; *p* = 0.389).

### 3.3. Participant Characteristics According to MRI-Derived Structural Profiles and Pain Status

Table 3 summarizes the demographic and occupational characteristics of the four subgroups defined according to MRI-derived structural profiles and the presence or absence of low back pain (LBP). Age was the only variable that differed significantly among the groups (*p* = 0.007), whereas no significant differences were observed for any other demographic or occupational characteristic (all *p* > 0.05). Participants younger than 40 years were predominantly classified within the disc–facet degenerative profile with LBP, whereas participants aged 50 years or older were more frequently classified within the same profile without LBP.

**Figure 2:**
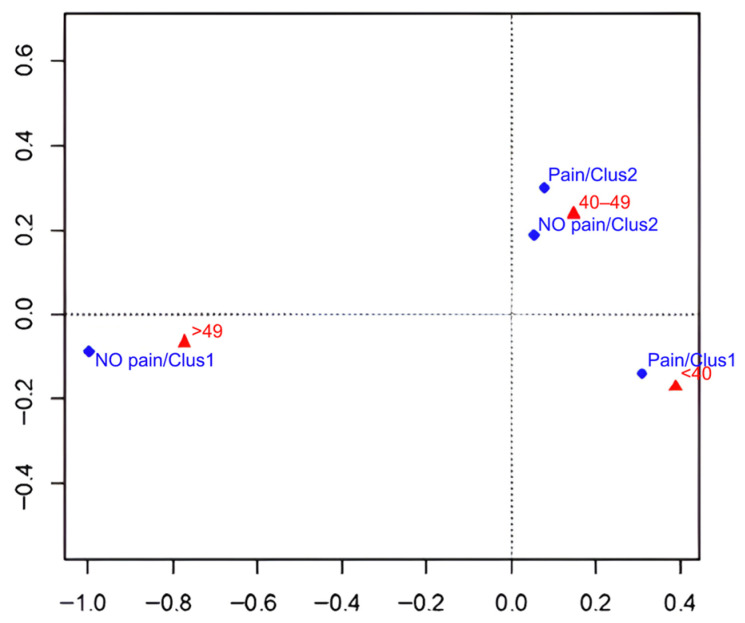
Age categories across MRI-based clusters with and without lumbar pain. The points represent the two identified clusters (Cluster 1: facet-degenerative profile; Cluster 2: disc profile without bony degeneration), differentiated by the presence or absence of pain (Pain/No pain) and by age categories (<40, 40–49, and >50 years).

Figure 2 shows the correspondence analysis between age categories and the four subgroups defined by MRI-based clusters and the presence or absence of pain. First, regarding younger and older participants, the figure clearly shows these relationships. In the right quadrant, the facet degenerative profile (Cluster 1) with pain is closely associated with participants under 40 years of age, suggesting that this younger subgroup tends to present more symptoms. In contrast, in the left quadrant, the facet degenerative profile (Cluster 1) without pain is associated with participants over 49 years old, indicating that, in this group, advanced age is not necessarily related to the presence of pain despite the existence of degenerative findings.

Secondly, the figure clarifies that the disc profile without osseous degeneration (Cluster 2), both in participants with and without pain, is located in the upper-central area, near the 40–49 age category. This pattern reflects that middle-aged participants are more evenly distributed within this structural profile.

## 4. Discussion

The present study identified two distinct lumbar MRI-derived structural profiles among actively practicing dentists: a disc–facet degenerative profile and a non-osseous degeneration profile. Although these profiles differed in their structural characteristics, neither was associated with the presence of low back pain (LBP) or pain-related disability. These results indicate that structural abnormalities identified by magnetic resonance imaging (MRI) alone are insufficient to explain pain presentation in this occupational group. This finding agrees with previous reports showing that degenerative MRI changes are frequently observed in both symptomatic and asymptomatic individuals. These findings further support the growing body of evidence indicating that lumbar degenerative changes detected by MRI should not automatically be regarded as the anatomical source of pain. Degenerative findings are highly prevalent in asymptomatic adults and tend to increase with age, suggesting that many structural abnormalities may represent age-related changes rather than clinically relevant pathology. Consequently, MRI findings should always be interpreted within the broader clinical context of each patient.

In agreement with previous evidence, no significant associations were found between individual MRI findings, including disc herniation, Modic changes, and annular fissure, and the presence of LBP [16,18,21]. By contrast, disc bulging, facet joint degeneration, and osteophytes were the variables that mainly distinguished the MRI-derived structural profiles. However, these differences in structural patterns were not reflected in differences in pain prevalence or disability. Collectively, these findings support the view that lumbar degenerative changes should be interpreted as part of a broader clinical assessment rather than as isolated predictors of low back pain. This observation is consistent with the current understanding of LBP as a multifactorial condition in which structural degeneration represents only one component of a complex clinical picture. Biomechanical, functional, psychosocial, and occupational factors are also known to contribute to symptom development and persistence. Therefore, relying exclusively on imaging findings may oversimplify the mechanisms underlying pain in healthcare professionals exposed to prolonged physical demands.

Age emerged as a factor potentially influencing the clinical expression of the identified MRI-derived profiles. Younger dentists were more frequently included in the disc–facet degenerative profile with LBP, whereas older participants presenting comparable structural changes were more commonly classified within the same profile without pain. The correspondence analysis reinforced this pattern, suggesting that age may have a greater influence on symptom manifestation than the degree of structural degeneration itself. Adaptation to occupational demands, cumulative professional experience, modifications in movement strategies, or differences in mechanical load tolerance could partly explain these findings, although longitudinal studies are needed to clarify these mechanisms.

Another noteworthy result was the lack of association between the MRI-derived structural profiles and occupational factors commonly linked to LBP, including working posture, weekly working hours, and chair support. Although ergonomic exposure has previously been identified as an important contributor to musculoskeletal disorders among dentists [7,8,9], these variables were not related to specific MRI-derived profiles in the present sample. This may indicate that occupational exposures contribute to pain mainly through functional or biomechanical mechanisms that are not necessarily accompanied by structural abnormalities detectable by MRI. Furthermore, MRI provides a static assessment of spinal morphology, whereas occupational exposures are dynamic and cumulative over time. Mechanical overload may induce transient muscular fatigue, altered movement patterns, or functional adaptations that are not necessarily accompanied by permanent structural abnormalities visible on conventional MRI. This may partly explain the lack of association between occupational variables and the identified structural profiles.

The present findings may have relevant clinical implications. The identification of MRI-derived structural profiles provides additional insight into the structural heterogeneity of lumbar degeneration within a relatively homogeneous occupational population. Furthermore, the different pain patterns observed between younger and older dentists despite similar MRI profiles underline the importance of considering age when interpreting lumbar imaging findings and when designing preventive or therapeutic interventions. From a clinical perspective, these findings emphasize the importance of adopting a comprehensive approach when evaluating dentists with LBP. Imaging findings should be integrated with clinical history, physical examination, and occupational assessment to avoid attributing symptoms exclusively to structural abnormalities. This approach may facilitate more individualized preventive and therapeutic strategies while reducing unnecessary concern regarding incidental degenerative MRI findings.

These findings may help clinicians avoid overinterpreting isolated MRI abnormalities when evaluating dentists with LBP. Clinical decision-making should integrate imaging findings with symptoms, physical examination, occupational exposure, and functional status. Such an approach may reduce unnecessary concern regarding incidental degenerative findings while supporting more individualized management strategies.

Several limitations should be acknowledged. First, spinal canal stenosis was excluded from the cluster analysis because of its low prevalence, allowing more robust cluster identification. Second, the modest sample size may limit the external validity of the findings and larger studies are required to confirm these results. Third, MRI examinations were interpreted by a single experienced musculoskeletal radiologist, preventing assessment of interobserver agreement. Finally, the cross-sectional nature of the study does not permit causal inference regarding the relationships among occupational exposure, lumbar MRI abnormalities, and low back pain. Future longitudinal studies are needed to determine the evolution and clinical relevance of the MRI-derived structural profiles identified in this investigation.

## 5. Conclusions

In conclusion, two distinct lumbar MRI-derived structural profiles were identified in actively practicing dentists: a disc–facet degenerative profile and a non-osseous degeneration profile. Despite their different structural characteristics, neither profile was associated with the presence of low back pain or pain-related disability. Age was associated with different pain status among dentists with the disc–facet degenerative profile, with younger professionals more frequently reporting pain than older professionals with similar structural findings. Overall, these findings highlight the limited explanatory value of lumbar MRI when interpreted in isolation and support the need for a broader, age-sensitive clinical approach to the assessment of low back pain.

## Figures and Tables

**Table 1:** Demographic, occupational, clinical, and MRI characteristics of the study participants according to low back pain status.

Variables	All (*n* = 57)	With Pain (*n* = 38)	Without Pain (*n* = 19)	*p* Value
**Demographic**				
Sex Female	31 (54.4%)	19(61.3%)	12(38.7%)	0.407
Age				
<40	22 (38.6%)	18 (81.8%)	4 (18.2%)	
40–49	20 (35.1%)	14 (70.0%)	6 (30.0%)	0.03
>50	15 (26.3%)	6 (40.0%)	9 (60.0%)	
**Professional practices**				
Length of Service	18.3 (±12.2)	18.0 (±10.9)	18.7 (±14.9)	0.147
Weekly working hours ≥ 36	28 (49.1%)	20(52.6%)	8 (42.1%)	0.576
Chair support:				
*Without* *support*	22 (38.6%)	13 (59.1%)	9 (40.9%)	0.114
*With 1 support*	19 (33.3%)	12 (63.2%)	7 (36.8%)	
*With 2 supports*	16 (28.1%)	13 (81.3%)	3 (18.8%)	
Working position:				
*Sitting*	21 (36.8%)	15 (71.4%)	6 (28.6%)	0.676
*Standing*	4 (7.0%)	2 (50.0%)	2 (50%)	
*Both*	32 (56.1%)	21 (65.6%)	11 (34.4%)	
**MRI measures**				
Annular fissure	12 (21.1%)	9 (23.7%)	3 (15.8%)	0.491
Disc bulging	48 (84.2%)	33 (86.8%)	15 (78.9%)	0.379
Disc herniation	21 (36.8%)	15 (39.5%)	6 (31.6%)	0.441
Spinal canal stenosis	6 (10.5%)	5 (13.2%)	1 (5.3%)	0.360
Modic changes	11 (19.3%)	9 (81.8%)	2 (18.2%)	0.235
Osteophytes	9 (15.8%)	4 (10.5%)	5 (26.3%)	0.123
Facet joint degeneration	38 (66.7%)	29 (76.3%)	9 (47.4%)	0.029
**Pain measures**				
ODI > 20	6 (10.5%)	6 (15.8%)	0 (0.0%)	0.067

Values are expressed as *n* (%) or mean ± standard deviation. Group comparisons were performed using chi-squared tests. MRI: magnetic resonance imaging; ODI: Oswestry Disability Index.

**Table 2:** Characteristics of MRI-based clusters.

MRI Measures	Cluster 1 (*n* = 38)	Cluster 2 (*n* = 19)	*p* Value
Fissure in the annulus fibrosus	11 (28.9%)	1 (5.3%)	0.450
Bulging disc	38 (100%)	10 (52.6%)	0.000
Herniated disc	17 (44.7%)	4 (21.1%)	0.144
Modic-type changes	9 (23.7%)	2 (10.5%)	0.304
Presence of osteophytes	9 (23.7%)	0 (0%)	0.022
Facet joint degeneration	36 (94.7%)	2 (10.5%)	0.000
**Pain measures**			
Pain	28 (73.7%)	10 (52.6%)	0.112
Oswestry score > 20	3 (7.9%)	3 (15.8%)	0.389

Values are expressed as *n* (%). *p* values were calculated using chi-squared tests. MRI: magnetic resonance imaging; ODI: Oswestry Disability Index.

**Table 3:** Participants’ characteristics related to MRI-based clusters with and without pain.

	Cluster 1 (*n* = 38)		Cluster 2 (*n* = 19)		
Demographic	Without Pain	With Pain	Without Pain	With Pain	*p* Value
Sex Female	5 (16.1%)	16 (51.6%)	7 (22.6%)	3 (9.7%)	0.220
Age					
<40	1 (4.5%)	15 (68.2%)	3 (13.6%)	3 (13.6%)	0.007
40–49	2 (10%)	9 (45.0%)	4 (20.0%)	5 (25.0%)	
>49	7 (46.7%)	4 (26.7%)	2 (13.3%)	2 (13.3%)	
**Professionals**					
Weekly Hours > 36 h	5 (17.2%)	13 (44.8%)	6 (20.7%)	5 (17.2%)	0.215
Chair Choice					
Without support	4 (18.2%)	9 (40.9%)	5 (22.7%)	4 (18.2%)	0.069
With 1 support	3 (15.8%)	9 (47.4%)	4 (21.1%)	3 (15.8%)	
With 2 supports	3 (18.8%)	10 (62.5%)	0 (0%)	3 (18.8%)	
Working position					
Sitting	4 (19.0%)	11 (52.4%)	2 (9.5%)	4 (19.0%)	0.320
Standing	2 (50.0%)	2 (50.0%)	0 (0%)	0 (0%)	
Both	4 (12.5%)	15 (46.9%)	7 (21.9%)	6 (18.8%)	

Values are presented as *n* (%). *p* values were calculated using chi-squared tests comparing the distribution of demographic and occupational variables across MRI-derived clusters according to pain status. MRI: magnetic resonance imaging.

## Data Availability

The data presented in this study are available from the corresponding author upon reasonable request. Public sharing of the dataset is restricted due to ethical approval requirements and the need to protect participant privacy and confidentiality.

## Abbreviations

The following abbreviations are used in this manuscript:

LBP	Low Back Pain
MRI	Magnetic Resonance Imaging
ODI	Oswestry Disability Index
SD	Standard Deviation
